# Shall We Dance? Recreational Dance, Well-Being and Productivity Performance During COVID-19: A Three-Country Study

**DOI:** 10.1177/1069031X221079609

**Published:** 2022-06

**Authors:** Michela Vecchi, Patrick Elf, Akiko Ueno, Athina Dilmperi, Charles Dennis, Luke Devereux

**Keywords:** subjective well-being, productivity, recreational dance, intrinsic motivation, self-determination theory, cultural tightness, social marketing

## Abstract

Mental health issues are increasingly prevalent worldwide, emphasizing the need to research antecedents and consequences of well-being. Prior research shows that within organizations, higher levels of subjective well-being (SWB) promote productivity performance. Building on this research, the authors hypothesize that recreational dance positively influences productivity through higher SWB. Survey data from Brazil, Italy, and the United Kingdom reveal that recreational dancers are more productive than nondancers due to their higher intrinsic motivation and SWB. Dancing has an additional direct effect on productivity, beyond the mediating role of SWB. The results indicate well-being and productivity improvements in all three countries, although they show a moderating effect such that the relationship between recreational dance and SWB is stronger when social norms are perceived to be looser. This study indicates potentially far-reaching benefits that could be achieved by including recreational dance in corporate well-being programs. International dance organizations could market dance classes as a pathway to increase productivity at work and explore synergies with public health marketing to promote the benefits of recreational dance in joint international campaigns.

Societies worldwide have experienced significant improvements in a wide range of areas, including increases in income, declining premature deaths, and improved longevity (Pinker 2018). However, despite these improvements, rising levels of stress and mental health issues have contributed to a decline in well-being. Recent studies have documented increasing levels of depression (Case and Deaton 2015; Rockett et al. 2016; Weinberger et al. 2018), which is now considered among the main causes of disabilities in Western societies (McManus et al. 2016; World Health Organization [WHO] 2013, 2016). Alarmingly, the proportion of adults experiencing symptoms of depression worldwide has greatly increased during the COVID-19 pandemic (Abbott 2021; Williams et al. 2021), which has posed further challenges for firms and organizations. From a marketing perspective, prior research has indicated that greater employee well-being leads to better performance, customer satisfaction, and safety (Fisher 2010), while lower levels can lead to poor decision making, absenteeism, presenteeism, and reduced productivity (Boyd 1997). Given the global scale of the current pandemic, international marketing strategies to improve subjective well-being (SWB)—defined as “a person's cognitive and affective evaluations of his or her life” (Diener, Oishi, and Lucas 2002, p. 63)—are of major importance.

In an attempt to tackle these growing mental health concerns, organizations worldwide have employed workplace interventions designed to improve employees’ health and SWB (Chartered Institute of Personnel and Development 2018). Similarly, many developed countries have introduced well-being and mental health social marketing strategies in public health policy over the past decade (e.g., Donovan and Henley 2010; Samele, Frew, and Urquia 2013) to promote mental health and reduce the economic burden that results from mental illness (Steptoe, Deaton, and Stone 2015; WHO 2013), which includes lower labor force participation and employment (Chatterji, Alegria, and Takeuchi 2011; Frijters, Johnston, and Shields 2014), lower educational attainment, and lower income and productivity (Ford et al. 2011; Johnston, Schurer, and Shields 2013).

Physical exercise is an effective way to fight mental illness and improve SWB. Although it is often associated with physical benefits, such as weight loss and improved heart conditions, research has shown that it also reduces anxiety and depression, and improves cognitive skills, resulting in better ability to memorize instructions, learn, perform complex tasks, and reason logically (Ratey 2008; Ratey and Loehr 2011). Dance as a form of physical exercise (recreational dance) is gaining ground among psychologists and neuroscientists because of its enhanced effects on several brain functions (Karpati et al. 2015). These researchers are increasingly recognizing that recreational dance not only has an entertainment purpose but can contribute to health and SWB (Courchesne, Ravanas, and Pulido 2017). Dance as a recreational activity requires observing and memorizing sequences of movements, moving in synchrony with others, and interpreting different roles, which stimulates a wider range of brain activities than other forms of physical exercise (Hanna 2015). Prior research has identified recreational dance as effective in relieving the symptoms of serious degenerative conditions such as dementia and Parkinson's disease and in enhancing well-being in old age (Houston and McGill 2013; Kattenstroth et al. 2013). However, despite these insights from neuroscience and psychology, which provide an increasingly robust body of evidence of the benefits of dance, few researchers have examined the marketing of dance and the relationship between dance and well-being in healthy individuals.

Improving well-being is in itself an important objective; higher levels of well-being hold the potential to influence several aspects of people's private and working lives. For example, recent research has shown that higher SWB can lead to productivity gains (Oswald, Proto, and Sgroi 2015), which suggests that recreational dance could promote productivity via increasing well-being. Although the recreational dance–well-being and well-being–productivity relationships have separately attracted much research over recent years, the recreational dance–productivity association requires further investigation.

Two additional factors can influence well-being and the recreational dance–well-being–productivity nexus—namely, individuals’ intrinsic motivation and the external environment where individuals operate. According to self-determination theory (SDT), individuals experience higher levels of SWB when their actions are the result of intrinsic motivation; that is, higher levels of SWB follow from doing an activity for its inherent satisfaction rather than for an external or extrinsic reward (Ryan and Deci 2017). Competence, autonomy, and connections with others are the key psychological aspects necessary to experience intrinsic motivation (Ryan and Deci 2000). This theoretical framework applies to several life domains, including dance, which is known to be a strongly intrinsically motivated activity (Maraz et al. 2015). Productivity can also be affected by intrinsic motivation, as the satisfaction of the key psychological needs plays an important role in improving both SWB and performance in the workplace (Manganelli et al. 2018; Ryan and Deci 2000). However, the question of whether intrinsic motivation can affect productivity via dance and well-being remains unanswered.

Lastly, the relationship between recreational dance, SWB and productivity can differ across countries. Advocates of cultural tightness–looseness theory (Gelfand, Harrington, and Fernandez 2017; Gelfand et al. 2011, 2020) have demonstrated that the set of social norms that operate within a culture, and the degree to which individuals abide by these norms, can affect well-being and productivity, such that a balance between freedom (looseness) and constraints (tightness) leads to higher levels of SWB and productivity. These views are particularly important during the current COVID-19 pandemic, in which countries have adopted various ways of tightening their rules to contain the spread of the virus and mortality rates. Accounting for cross-cultural differences in social norms in terms of tightness–looseness theory can provide important insights for international marketing strategies to promote well-being in a particularly challenging situation.

Building on contributions from marketing, psychology, economics, and neuroscience, this multidisciplinary study investigates the relationship between recreational dance, SWB, and productivity performance within an international framework. To the best of our knowledge, no extant studies address the relationship between dance, well-being, and productivity, nor are there suitable data sets that would allow exploring the interplay between dance practice, well-being, and productivity. This study is the first to gather the necessary data for three countries (the United Kingdom, Italy, and Brazil) using a survey of working adults who attend dance classes as a recreational activity. Our results provide support for the positive relationship between recreational dance and SWB in all countries, although we find a moderating effect such that the relationship is stronger where social norms are perceived to be looser. In addition, using individuals’ assessments of their performance at work, we conclude that recreational dance, and the intrinsic motivation to dance, influences productivity performance indirectly through well-being and directly beyond the mediated effect of SWB.

Our study provides an important contribution to productivity and well-being theory by establishing dance practice as a contributor to productivity performance. Our results support the introduction of recreational dance in corporate well-being programs. Dance organizations could promote the benefits of recreational dance in a variety of countries (even accounting for the moderating effect of perceived social norms). The international dimension of our study sheds light on how social norms lead to higher levels of well-being and productivity and can inform international marketers when promoting dance as a recreational activity worldwide. Such international marketing strategies are likely to achieve better productivity outcomes in countries characterized by tighter social norms. Our study also extends knowledge in the field of self-determination theory by associating individuals from tighter cultures with higher intrinsic motivation and by confirming the well-established positive association between intrinsic motivation and well-being. This finding can inform policy makers, businesses, and dance organizations in designing international marketing communications linking the psychological need to experience intrinsic motivation with SWB, recreational dance, and productivity. Finally, results from our analysis have implications for public health marketing, suggesting the diffusion of recreational dance classes worldwide to improve consumers’ SWB and their performance in the workplace during a health crisis.

The remainder of this article is structured as follows. We begin by reviewing the literature on the importance of recreational dance for well-being and the link between well-being and productivity. We then summarize the main predictions of self-determination and cultural tightness–looseness theories in relation to the objective of our study. Building on our literature review, we introduce our theoretical framework and hypothesis development. Next, we present details of our data collection and an overview of our analytical methods. We next discuss our findings and their implications for dance organizations, international marketers, and policy makers. Finally, we address limitations and suggest several avenues for future research.

## Literature Review and Hypothesis Development

### Exercise, Dance, and Well-Being

Central to the argument presented herein is the idea that physical exercise has a positive effect on well-being. Research exploring this relationship has received much attention over the years, consistently showing that physical activity positively impacts health and well-being (Colcombe and Kramer 2003; Hassmén, Koivula, and Uutela 2000; Ratey and Loehr 2011; Scully et al. 1998). Studies have shown that physical activity helps with a variety of disorders such as anxiety (Stubbs et al. 2017), stress (Gerber and Pühse 2009; Wyss et al. 2016), and stress-related disease (Gerber and Pühse 2009; Silverman and Deuster 2014). These positive results have been observed across age groups, from children (Li et al. 2021) to the elderly (Zubala et al. 2017). Complementing these findings, other studies have linked physical fitness with higher levels of resilience (Silverman and Deuster 2014), which, in turn, positively affects well-being (Goodman et al. 2017).^1^

Recent evidence shows that, compared with other exercise practices, recreational dance provides additional benefits due to the complex brain functions activated when dancing. Dance combines the benefits of music (Franco et al. 2014; Tierney and Kraus 2013), which stimulates the reward center of the brain, with brain regions that are associated with motor, sensor, and coordination functions. Thus, when people dance different parts of the brain communicate with each other, improving memory, empathy, and emotional intelligence, while reducing stress levels (Hanna 2015). Recreational dance is also particularly effective in preventing cognitive decline in older individuals (Kattenstroth et al. 2013; Rehfeld et al. 2017), including those suffering from degenerative conditions like Parkinson's (Houston and McGill 2013). Crucially, Rehfeld et al. (2017) find that regular physical exercise can reverse the signs of aging in older people's brains, and dancing has the largest effects compared with not only other forms of exercise but also cognitive activities such as reading books, writing for pleasure, and crossword puzzles.

Moreover, the positive effects of recreational dance on cognition and well-being are not confined to old age. For example, Muro and Artero (2016) found improvements in Satisfaction with Life Scale scores in young women attending three dance schools in Barcelona. Studies have also investigated the effect on well-being of different types of dance such as tango (Kreutz 2008; Pinniger et al. 2012), circle dance (Borges da Costa and Cox 2016), and West African dance (Conner, Patterson-Price, and Faulkner 2021). Results from these studies confirm the positive effects of recreational dance on relieving stress, improving relaxation, and overall well-being. Considering that dance practices are important cultural assets in all countries, the potential benefits could be global. Table W1 in Web Appendix A summarizes the results of several studies, including several review articles.

### The Influence of Well-Being, Mental Health, and Recreational Dance on Productivity Performance

While achieving higher levels of well-being is an important objective per se, it can also have consequences on individuals’ behavior in the workplace and on their productivity.^2^ Improving productivity performance has been at the center of the economic and political debate in many countries, particularly after the 2008 financial crisis (e.g., Barnett et al. 2014; Pessoa and Van Reenen 2014). Central to this discussion is the question of which factors could promote productivity. In addition to the traditional drivers of productivity growth, such as innovation activities (Griffith, Redding, and Van Reenen 2004; O’Mahony and Vecchi 2009), management practices (Bertschek and Kaiser 2004; Bloom and Van Reenen 2011), and investments in human capital (Black and Lynch 1996; Mason, O’Leary, and Vecchi 2012), the role of well-being has received close attention (Clark 2018).

Studies have shown that higher levels of well-being are correlated with greater workers’ productivity (DiMaria, Peroni, and Sarracino 2019; Isham, Mair, and Jackson 2020). Going beyond a positive correlation, a study by Oswald, Proto, and Sgroi (2015) involving four randomized control trials shows that young men and women at an English university who were exposed to a “happiness treatment” were approximately 12% more productive compared with the benchmark, thus providing support for a causal link between well-being and productivity. Earlier related work by Tsai, Chen, and Liu (2007) also shows that positive mood, a proxy for well-being, predicts task performance. It is important to note that the theoretical mechanisms that lead to the positive relationship between well-being and productivity differ across studies. For example, the model of Oswald, Proto, and Sgroi (2015) is based on distracted worrying, whereby an individual who is exposed to a positive happiness shock devotes more attention and effort to solving problems at work and less attention to worrying about other things. In contrast, Tsai, Chen, and Liu (2007) refer to interpersonal processes, finding that higher levels of well-being induce helping behavior toward coworkers, which increases productivity.

Another mechanism that leads to a positive relationship between well-being and productivity is physical and mental health. Poor physical and mental health are associated with lower levels of well-being, which can cause productivity losses either because workers are absent from work (absenteeism) or because they are not able to fully perform at work due to health conditions (presenteeism). Presenteeism is particularly common among those facing mental health issues (Ford et al. 2011; Isham, Mair, and Jackson 2020), and the costs related to lost productive work time due to presenteeism are substantial: in 2001, these costs were estimated at US$44 billion per year in the United States (Stewart et al. 2003); more recent evidence for the United Kingdom estimates that the cost of presenteeism to businesses is double that of absenteeism (Parsonage and Saini 2017).

The discussion so far has highlighted the importance of improving well-being to promote productivity performance within businesses. Given the strong evidence documenting the positive effect of recreational dance on well-being, dancing could be an effective way to contribute to higher productivity via the mediated effect of well-being. Moreover, recreational dance could have an additional impact on productivity, unmediated by well-being due to its documented effect on cognitive functions (Earhart 2009; Kattenstroth et al. 2013; Rehfeld et al. 2017; Teixeira-Machado and Coutinho 2018), that is, the ability to memorize instructions, learn and perform complex tasks, and reason logically. Across the economic literature, research has traditionally related cognitive skills to productivity, as highlighted in the seminal contributions by Becker (1975) and Mincer (1975) as well as in more recent contributions (e.g., Cawley, Heckman, and Vytlacil 1999, 2001; Heckman, Stixrud, and Urzua 2006). Studies have also documented that cognitive skills naturally decline with age, resulting in poorer performance in everyday tasks (Whitley, Deary, and Ritchie 2016). However, no studies examine these important connections in relation to recreational dance, which makes our analysis particularly compelling.

### Well-Being and Intrinsic Motivation

An important theoretical approach to the study of human behavior complementing our theoretical framework is SDT, which provides an empirically validated method to examining factors that promote intrinsic motivation and well-being. According to SDT, people experience intrinsic motivation when their engagement in an activity is the result of personal interest (Ryan and Deci 2000, 2017). A key proposition is that to experience intrinsic motivation, individuals need to feel competent in a particular life context, connected to others, and able to take autonomous decisions (Deci and Ryan 2000; Ryan and Deci 2017). In other words, people who feel more autonomous, competent, and related to others experience higher levels of well-being (Kasser et al. 2014).

Although SDT's importance in the study of well-being is demonstrated by far-reaching evidence obtained through extensive research in a number of areas and life domains (Deci and Ryan 2008), including physical exercise (Gunnell et al. 2014; Sebire, Standage, and Vansteenkiste 2009), these studies do not encompass recreational dance. Some evidence shows that dance is a highly intrinsically motivated activity (Kreutz 2008), but only two studies refer to SDT in the analysis of dancers’ motivations. First, Maraz et al. (2015) report that, while dancing shares similar motivational factors with other activities such as exercise and gambling, self-confidence and intimacy appear to be specific to dancing. In addition, mood enhancement and stress reduction are important motivational factors among dancers. Second, Sebire et al. (2016) implemented SDT in the design of an experiment aimed at promoting recreational dance among adolescent girls. Despite the lack of effectiveness of the intervention, the theoretical approach proved extremely useful in the evaluation of physical activity interventions. Thus, evidence of the relationship between intrinsic motivation and dance is scant and requires further investigation.

Finally, another life domain in which SDT provides an important contribution is the workplace (Gomez-Baya and Lucia-Casademunt 2018; Roney and Soicher 2021). Research has shown that the satisfaction of the three basic psychological needs has important consequences for individuals’ work, as intrinsic motivation promotes well-being and performance in the workplace (Manganelli et al. 2018; Rigby and Ryan 2018).

### Tight and Loose Social Norms and Well-Being

Individuals operate within their social and cultural environments, which are characterized by unique (social) norms that inform individuals’ behavior and can affect well-being. Gelfand et al. (2011) introduce a continuum from tight to loose, whereby tight cultures are characterized by strong social norms with a low tolerance for deviant behaviors, while loose cultures have weaker social norms and are considered more tolerant of deviant conduct (Gelfand 2012; Gelfand et al. 2011; Madan et al. 2018; Seitz et al. 2020). Tightness and looseness can vary at the national, local, and individual levels (Gelfand et al. 2011; Madan et al. 2018), offering a useful framework to describe and analyze individuals’ behaviors as well as business, marketing strategies, and economic outcomes (Harrington, Boski, and Gelfand 2015).

The relationship between different social norms and well-being is complex, and the debate over whether freedom or rules lead to higher levels of well-being in societies goes back to ancient Greece (Gelfand et al. 2011). In practice, research shows that a moderate situation maximizing both openness and order is most likely to result in increased levels of well-being within societies. For example, Harrington, Boski, and Gelfand (2015) show that the relationship between well-being and tightness is U-shaped: extremely loose countries (i.e., Brazil, Greece, and Hungary) and extremely tight cultures (i.e., Pakistan and India) report lower levels of well-being compared with intermediate countries (i.e., Italy, France, and the United Kingdom). Conversely, the relationship between social norms and creative activities, such as dance, is less clear-cut: although the general view is that cultural tightness inhibits creativity (Gelfand, Nishii, and Raver 2006), whether an intermediate situation along the tight-loose spectrum could be beneficial for creativity is largely unknown.

Lastly, studies also indicate that preferences for tight or loose social norms can be altered in situations of crisis (Seitz et al. 2020). This follows the notion that stronger norms and active opposition of deviant behavior promote cooperation, which leads to a more rapid response to external threats (Gelfand, Harrington, and Fernandez 2017; Roos et al. 2015). For instance, early responses during the initial stages of the COVID-19 pandemic varied greatly between nations, wherein some loose societies (e.g., Brazil) showed a delayed and often conflicted reaction to tightening norms (Seitz et al. 2020). As nations inevitably tightened in an attempt to coordinate actions to combat the virus, tighter cultures (e.g., Japan) were more readily equipped to make swift responses (Seitz et al. 2020). Indeed, Gelfand et al. (2021) report that tighter cultures experienced lower rates of COVID-19 infection and a lower number of deaths than looser cultures.

The introduction of long lockdown periods has decreased well-being across the world (Brodeur et al. 2021). Whether this effect has been stronger in tighter or looser cultures is difficult to predict a priori and needs further empirical investigation. With regard to the link between the global health pandemic and recreational dance, COVID-19 has disrupted dance practices globally (Tariao and Yang 2021), often preventing people from enjoying the well-being benefits provided by these activities. Although online delivery of classes has become increasingly popular, movements are restricted to small spaces, and few opportunities are available for feedback and learning of advanced movements in online classes (Coelho and Menon 2020). Therefore, the relationship between dance and well-being may have weakened during the pandemic. As for workplace productivity, to our knowledge no studies compare tight versus loose cultures, while accounting for individuals’ well-being, in context of analyzing productivity performance. Thus, our analysis provides valuable new insights that go beyond the current health crisis.

## Hypothesis Development

**Figure 1.**
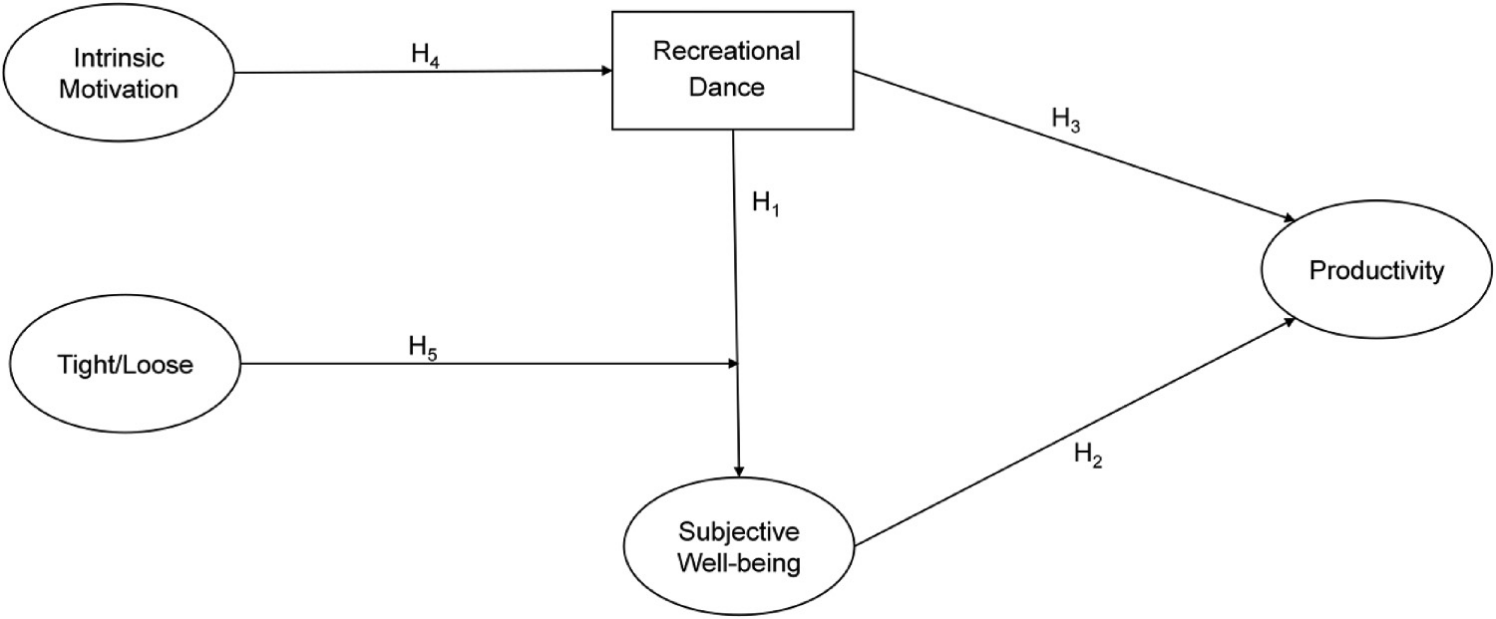
Conceptual model.

The theoretical underpinning of our study is multidisciplinary and relies on psychology, neuroscience, dance and economic literatures, as discussed in our literature review. Drawing from a broad body of evidence, Figure 1 presents our conceptual model, with its paths and hypotheses. We have located SWB at the center of our construct to act as the mediator between various factors and productivity performance (i.e., the dependent variable). The first factor we consider is dance performed at the recreational level. Our first hypothesis is that recreational dancers experience higher levels of well-being, given the evidence showing the positive effect of recreational dance on well-being (Borges da Costa and Cox 2016; Houston and McGill 2013; Kattenstroth et al. 2013; Pinniger et al. 2012):

**H_1_:** Recreational dancers are more likely to experience higher levels of SWB than nondancers.

Our second hypothesis stems from contributions from the economics literature, which documents the positive impact of SWB on productivity performance (Clark 2018; DiMaria, Peroni, and Sarracino 2019; Ford et al. 2011; Isham, Mair, and Jackson 2020; Oswald, Proto, and Sgroi 2015):

**H_2_:** Workers with high (low) levels of SWB are likely to be more (less) productive in the workplace.

H_1_ and H_2_ imply that recreational dance has an indirect effect on productivity, mediated by well-being. Other studies have considered well-being as a mediator. For example, Imran and Shahnawaz (2020) explore how well-being mediates the link between psychological capital and performance. Opree, Buijzen, and Van Reijmersdal (2016) argue that psychological well-being mediates the advertising effect on life satisfaction in children.

While H_1_ and H_2_ aim to extend the generalization of two well-known relationships, the next hypothesis aims to test the presence of a new, direct impact of recreational dance on productivity, beyond the mediated effect of well-being:

**H_3_:** Recreational dancers are more productive than nondancers in the workplace (direct effect, independent of their level of SWB).

This hypothesis stems from the contribution of two fields, psychology and neuroscience, which both demonstrate that dancing for recreational purposes has important effects on cognition (Earhart 2009; Karpati et al. 2015; Kattenstroth et al. 2013; Rehfeld et al. 2017), as well as the economic literature relating productivity and cognitive skills (Cawley, Heckman, and Vytlacil 1999; Heckman, Stixrud, and Urzua 2006; Lindqvist and Vestman 2011). In our model, both well-being and recreational dance act as mediators of the effect of intrinsic motivation on productivity. Evidence suggests that recreational dancers are highly motivated individuals and are driven by additional motivational factors compared with those who engage in other types of activities, including other types of physical exercise (Kreutz 2008; Maraz et al. 2015; Sebire et al. 2016). Thus, we expect recreational dancers to be more able to satisfy their psychological needs in line with SDT:

**H_4_:** Recreational dancers are more likely to be intrinsically motivated than nondancers.

Our theoretical framework also accounts for the moderating role of the cultural environment on the relationship between dance and well-being. Following the prevalent view that tight cultures have a negative impact on creativity, we expect that recreational dance is more relevant to the experience of well-being in loose cultures (Chua, Roth, and Lemoine 2015; Gelfand, Nishii, and Raver 2006). Given that our study was carried out in the midst of the COVID-19 pandemic, when tight rules have often made recreational dance difficult or impossible, we expect that where social norms are weaker, recreational dance will have a more positive effect on well-being. Therefore,

**H_5_:** Perceived looseness (tightness) of social norms moderates the relationship between recreational dance and SWB such that the relationship is stronger (weaker) when those norms are perceived as looser (tighter).

Currently, no research provides insights on the relationship between recreational dance and well-being in periods characterized by a sudden tightening of social norms; thus, testing this hypothesis will further our understanding of this important nexus.

Finally, extensive previous research confirms that individuals with higher intrinsic motivation experience greater levels of SWB (e.g., Deci and Ryan 2000; Kasser et al. 2014). This effect is well known and not central to our theory, so we do not offer a formal hypothesis to capture this relationship. Nonetheless, to ensure that our model fits the data realistically, we, include that path in our operationalized model.

## Methodology

### Content of Questionnaire

To test the hypotheses discussed in the previous section, we collected data from dancers (target group) and nondancers (control group) through an online questionnaire using Qualtrics as the survey platform. The content of the questionnaire was drawn from survey items used in previous research, which have been demonstrated to provide reliable scales. The main part of the questionnaire consisted of items on SWB, productivity (PROD), intrinsic motivation (IM), and perceptions of tight and loose social norms (T/L). We measured SWB using a five-item scale reflecting the extent to which respondents felt fulfilled with their lives. The scale has been psychometrically validated in previous studies (Dennis et al. 2016; Papagiannidis et al. 2017), and similar scale items have been used in related contributions (e.g., Hicks, Tinkler, and Allin 2013; Waldron 2010).

We adapted scale items for PROD from the Health and Work Performance questionnaire (HPQ), developed by the WHO (Kessler et al. 2003; Kessler and Üstün 2004). This questionnaire has been used for the analysis of the relationship between health and performance in several studies (Okazaki et al. 2019; Pronk et al. 2004; Wang et al. 2003). Kessler and Üstün (2004) document the excellent reliability, validity, and sensitivity to change of the HPQ measures. The questionnaire provides an evaluation of both presenteeism and absenteeism; however, given that well-being is particularly associated with presenteeism (Ford et al. 2011; Isham, Mair, and Jackson 2020), we use only presenteeism items in the current study. These measures capture the perceived quality of work, the level of concentration and care, and time spent not working while in the workplace. We changed the questionnaire slightly to focus primarily on work performance and to account for the different working conditions during the COVID-19 crisis. We added three further questions to capture the nature of the tasks performed in the workplace (manual/cognitive tasks, routine/nonroutine tasks, and group/individual work), in addition to information on the respondents’ occupations (classified according to the U.K. Standard Occupational Classification) and the industry in which respondents performed their main job (following the U.K. Standard Industrial Classification of economic activities).

For IM, we used a shortened version of the revised Motives for Physical Activities Measure scale (Ryan et al. 1997). The 15-item scale allowed us to identify motivations for why people engage in physical activities, including the fundamental needs of intrinsic motivation as well as other motivational factors such as fun and happiness. We changed the wording of the scale slightly for the dancers’ group so that questions referred to dance rather than to physical activities more generally.

Finally, we measured T/L using a total of six items reflecting respondents’ perceived norms in their countries of origin, adapted from Gelfand et al.’s (2011) supporting materials and Gelfand et al. (2020). The scales for WB, IM, and T/L used seven-point Likert-type response format (1 = “strongly disagree,” and 7 = “strongly agree”). Question items for PROD used a five-point scale ranging from 1 (“all of the time”) to 5 (“none of the time”) (for a summary of the indicators used in the study, see Web Appendix B).

### Target Samples and Data Collection

We recruited two samples, recreational dancers (target group) and nondancers (control group). Because gathering data from the whole target population was not feasible, we collected data for the dancer sample following a nonprobability convenience sampling method (Langer 2018). We collected data from dancers who were both willing and available to complete the survey. We imposed no other criteria apart from undertaking dance on the respondents. Convenience sampling is a widely used technique for consumer surveys (e.g., Dennis et al. 2018; Sharma, Ueno, and Kingshott 2021) because data can be collected quickly, straightforwardly, and inexpensively (Cooper, Schindler, and Sun 2006; Malhotra 2019). We collected data from dance organizations, a university's Faculty of Arts and Creative Industry, and social media platforms such as Twitter, Facebook, and Instagram. Three dance organizations agreed to disseminate the online questionnaire to their members who were located in several countries across the world.

We received the most responses from the United Kingdom, Italy, and Brazil, so we ultimately chose these countries for our analysis. We removed respondents who worked as dance professionals and those who failed to answer the PROD questions. All dancers included in the analysis are either employed or self-employed, outside the dance profession. The final sample used for the analysis includes 238 respondents from the United Kingdom, 172 from Italy, and 127 from Brazil, for a total of 537 observations for recreational dancers. We next used the same three countries to collect data from nondancers. This control group allowed us to compare the main outcome variables of our study (i.e., well-being and productivity) between recreational dancers and nondancers, thereby providing a thorough evaluation of the role of recreational dance. We collected all control group data through an external market research agency to ensure matched background information. The recruitment took place shortly after the data collection for the dance group to minimize changes in general circumstances that might have affected the outcome of our analysis (e.g., changes to lockdown rules and other social distancing measures). We disregarded partially completed questionnaires in our analysis. We collected a total of 956 completed responses for nondancers, with 275 from the United Kingdom, 325 from Italy, and 356 from Brazil. The main part of the questionnaire, including the sections on WB, PROD, IM, T/L, and demographics, was the same for nondancers.

Web Appendix C summarizes the main characteristics of recreational dancers and control group. The proportion of recreational dancers is highest in the United Kingdom (46.39%), followed by Italy (34.61%) and Brazil (26.29%) As we expected, the majority of respondents were female (87.45%), as recreational dance as a form of physical activity is more common among women. Most respondents were between 18 and 24 years of age, with a higher proportion of older respondents (aged 60 years and above) among recreational dancers (14.5%) compared with the control group (7.9%). The majority of respondents in both groups are more educated than their respective national averages, although the proportion of those with tertiary education is higher among recreational dancers (56.4%) than nondancers (46.8%).

### Pilot Testing

Before piloting the questionnaire, several academics with expertise within the main topics of the survey (i.e., WB, PROD, IM, and T/L), and in dance critically evaluated all scales. We incorporated their feedback into the questionnaire before running a pilot test to ensure that the questions were comprehensible and the overall questionnaire was easy to complete. A total of 132 completed questionnaires were collected within 10 days and subsequently analyzed using exploratory factor analysis and reliability testing using SPSS version 25, to determine those items that loaded together most strongly and identify items for deletion. Initially, the questionnaire was written in English. After the pilot, native speakers translated the questionnaire into Italian and Portuguese using Douglas and Craig’s (2007) collaborative and iterative method. Then, each translated questionnaire was evaluated by several academics whose first language was either Italian or Portuguese, following a back-translation method (Brislin 1970). That is, we modified the questionnaire following the first group's feedback before running additional pretests with a second set of researchers proficient in the respective language and English, to ensure that all the items corresponded to the English version. We excluded all pilot data from the sample used for the final analysis.

### Main Survey

The original IM scale had 15 items, which is more than what is required for a latent variable and, if all were used in structural equation modeling (SEM), would detract from the model's statistical power (Koran 2020). Therefore, the main study used a more parsimonious, reduced bank of items (eight after eliminating those with lower loadings in the pilot) for IM; this set of items still reflected the fundamental needs of intrinsic motivation (Goldman, Goodboy, and Weber 2017). We confirmed our choice of indicators with confirmatory factor analysis (CFA). In the main study, the removal of the seven items from SD due to low convergent validity helped us achieve desired discriminant validity. For SWB, we observed no change to the scales. We removed one item from the PROD scale and two items from the T/L scale in the main survey due to low convergent validity, such that we used a total of four items for each for data analysis. We tested these scales with CFA using IBM SPSS Amos 27 using the data. The results indicated reliability and convergent validity of the items, and appropriate correlations between latent variables, consistent with the hypothesized paths (Web Appendices B and D).

Before evaluating the hypothesized model, we used multiple regression analysis to investigate the possible effects of the control variables age, education, gender, and employment type (occupation and industry) on all indicators of well-being and productivity. These control variables made no significant difference to the models. In the interest of clarity, we therefore omitted the control variables from the SEM, which produced results consistent with the regression analysis. We tested the hypothesized paths using SEM, with IBM SPSS Amos 27. We tested the moderation hypothesis, H_5_, using a two-stage approach, which is appropriate for a continuous moderator, wherein the moderation term (the product of factor scores of T/L and the Recreational dancers terms) forms an independent variable alongside predictor and moderator variables (Fassot, Henseler, and Coelho 2016).^3^

## Results

Table 1 presents differences in the average scores between recreational dancers and nondancers for all indicators used in our analysis. We found that SWB is significantly higher for recreational dancers on four of the five multi-item indicators (Table 1, Panel A). Intrinsic motivation (Panel B) and productivity (Panel C) are significantly higher for recreational dancers compared with nondancers on all five indicators. Perceptions of tight and loose culture are similar for recreational dancers and nondancers, except that nondancers perceived that “people comply with social norms” significantly more than dancers (Panel D).

**Table 1.** Comparisons of Recreational Dancers and Nondancers According to Four Indicators.

Indicator	Nondancers Mean	Recreational Dancers Mean	Difference
**A: Well-Being Indicators**			
Life close to ideal	3.99	4.39	−.39***
Conditions of life excellent	4.10	4.42	−.32***
I am satisfied with my life	4.30	4.63	−.33***
I have the important things in life	4.81	4.93	−.12
If I could live my life over, I would change almost nothing	3.90	4.07	−.17*
**B: Intrinsic Motivation Indicators**			
I exercise/dance because			
I want to be physically fit	5.26	5.74	−.48***
It is fun	4.69	6.34	−1.65***
I like to engage in activities physically challenging	4.54	5.80	−1.26***
I want to learn new skills	4.85	5.99	−1.14***
I want to improve my skills	5.04	6.13	−1.09***
I like the challenge	4.73	5.74	−1.01***
It makes me feel happy	5.03	6.72	−1.69***
It is interesting	4.89	6.29	−1.40***
**C: Productivity Indicators**			
In the past week how often			
Did not work when supposed to?	3.61	4.09	−.48***
Work was less careful	3.57	3.94	−.37***
Quality was lower	3.84	4.17	−.33***
Not concentrated enough	3.62	3.95	−.33***
Health limited work	3.88	4.30	−.42***
**D: Tight–Loose Social Norms Indicators**			
Clear expectations of how people should act	4.44	4.35	.09
Many social norms that people are supposed to abide	4.77	4.88	−.11
People agree upon appropriate behavior	4.45	4.34	.11
People comply with social norms	4.05	3.88	.17**

*p < .1.

**p < .05.

***p < .01.

*Notes*: Panels A and B: seven-point scale anchored by 1 = “strongly disagree” and 7 = “strongly agree.” Panel C: five-point scale anchored by 1 = “all the time” and 5 = “none of the time.” Panel D: seven-point scale anchored by 1 = “strongly disagree” and 7 = “strongly agree” (i.e., tight is high, and loose is low).

Table 2 reports average values of all indicators, together with differences between recreational dancers and nondancers, for each country. Results for T/L indicate statistically significant differences across countries, with the United Kingdom (mean 4.46 on the 7-point scale) scoring significantly tighter than Brazil (4.24). Our observations are consistent with Gelfand et al. (2011), who report that the United Kingdom has the tightest culture, closely followed by Italy, while Brazil is positioned toward the looser end of the spectrum. In our findings, Italy (4.28) is not significantly different from Brazil (F(1, 231), n.s.), whereas the United Kingdom is significantly tighter than both Brazil (F(1, 42.0), *p* < .001) and Italy (F(1, 51.1), *p* < .001). This result is consistent with anecdotal evidence concerning how these countries handled the COVID-19 pandemic: Italy has been reported (albeit in nonverifiable sources) as loose, whereas the United Kingdom has been reported as medium (Beilby 2020).

**Table 2.** Differences in Well-Being, Productivity, and Intrinsic Motivation Between Recreational Dancers and Nondancers: Cross-Country Comparison.

	Tight (High) Versus Loose	Well-Being (Recreational Dancers Are on Average Higher)	Productivity (Recreational Dancers Are on Average Higher)	Intrinsic Motivation (Recreational Dancers Are on Average Higher)
Brazil	4.24	.370 (d = .301)	.188 (d = .238)	.90 (d = .875)
Italy	4.28	.162 (d = .130)	.259 (d = .335)	1.13 (d = 1.274)
United Kingdom	4.46	.447 (d = .358)	.507 (d = .639)	1.51 (d = 1.396)

*Notes*: d = Cohen’s d effect size. All measurements on a seven-point scale.

In all three countries, mean scores for well-being and productivity are higher for recreational dancers than nondancers. The differences between recreational dancers and nondancers are greater in the United Kingdom (well-being: Cohen's d effect size = .358; productivity: d = .639) than in Brazil (well-being: d = .301; productivity: d = .238) and Italy (well-being: d = .130; productivity: d = .335). The final column in Table 2 shows differences in intrinsic motivation between recreational dancers and nondancers within each country. Consistent with our expectations, recreational dancers are more intrinsically motivated than nondancers, with those in the United Kingdom reporting the largest difference (1.51), followed by Italy (1.13) and then Brazil (.90).

### Hypothesis Testing

We performed CFA and found a satisfactory fit (χ^2^ = 858, d.f. = 224; normed χ^2^ = 3.83; goodness-of-fit index [GFI] = .921; adjusted goodness of fit index [AGFI] = .905; root mean square error of approximation [RMSEA] = .055). We established convergent reliability, with all items loading greater than .5 (except one greater than .6) and construct reliability greater than .8 for each construct (Web Appendix B). We also established discriminant validity, as the square root of the average variance extracted for each of the constructs is greater than the correlations between them (Web Appendix D).

We used SEM to examine the relationships between the hypothesized constructs, with dancer (1) or not (0) included as a dummy variable (Table 3), because SEM allows us to simultaneously examine the relationships among the measured and latent constructs. This model yielded a satisfactory fit (χ^2^ = 948, d.f. = 223; normed χ^2^ = 3.90; GFI = .930; AGFI = .910; RMSEA = .060). We thus conclude that recreational dance has a positive effect on well-being (H_1_). Our data further show that well-being has a positive effect on productivity (H_2_), which also confirms that dance has an indirect effect on productivity via the well-being channel. In addition, our results confirm that recreational dance has a direct positive effect on productivity (in addition to the indirect effect via well-being) (H_3_) and that higher intrinsic motivation increases the likelihood that a person is a recreational dancer (H_4_).

**Table 3.** Structural Equation Model.

Path	Standardized Coefficient	t-Value
Intrinsic motivation → Recreational dance	.708	20.4****
Intrinsic motivation → Well-being	.119	2.3*
Tight/Loose → Well-being	.260	6.1****
Recreational dance → Well-being	.173	3.9****
Tight/Loose → Productivity	.198	5.5****
Recreational dance → Productivity	.097	3.3****
Well-being → Productivity	.106	2.6***
Moderation term → Well-being	−.110	−2.9***

**p ≤ .05.

***p ≤ .01.

****p ≤ .001.

*Notes*: Estimation: asymptotic distribution free.

The moderation term for the effect of perceived tightness/looseness of social norms on the relationship between recreational dance and SWB is significant, such that the relationship is stronger when those norms are perceived as looser (H_5_). The moderation evaluation necessarily includes examining the direct effect of the moderator (T/L) on SWB, and we observed that perceptions of tighter social norms positively affect SWB. As discussed in our literature review, preferences for tight rules tend to prevail in situations of crisis (Seitz et al. 2020). Given that the study has been carried out in the midst of the COVID-19 pandemic, our results are consistent with recent contributions from the tight and loose theory, whereby tighter norms promote a faster response to the challenges imposed by external threats (Gelfand et al. 2021).

**Figure 2.**
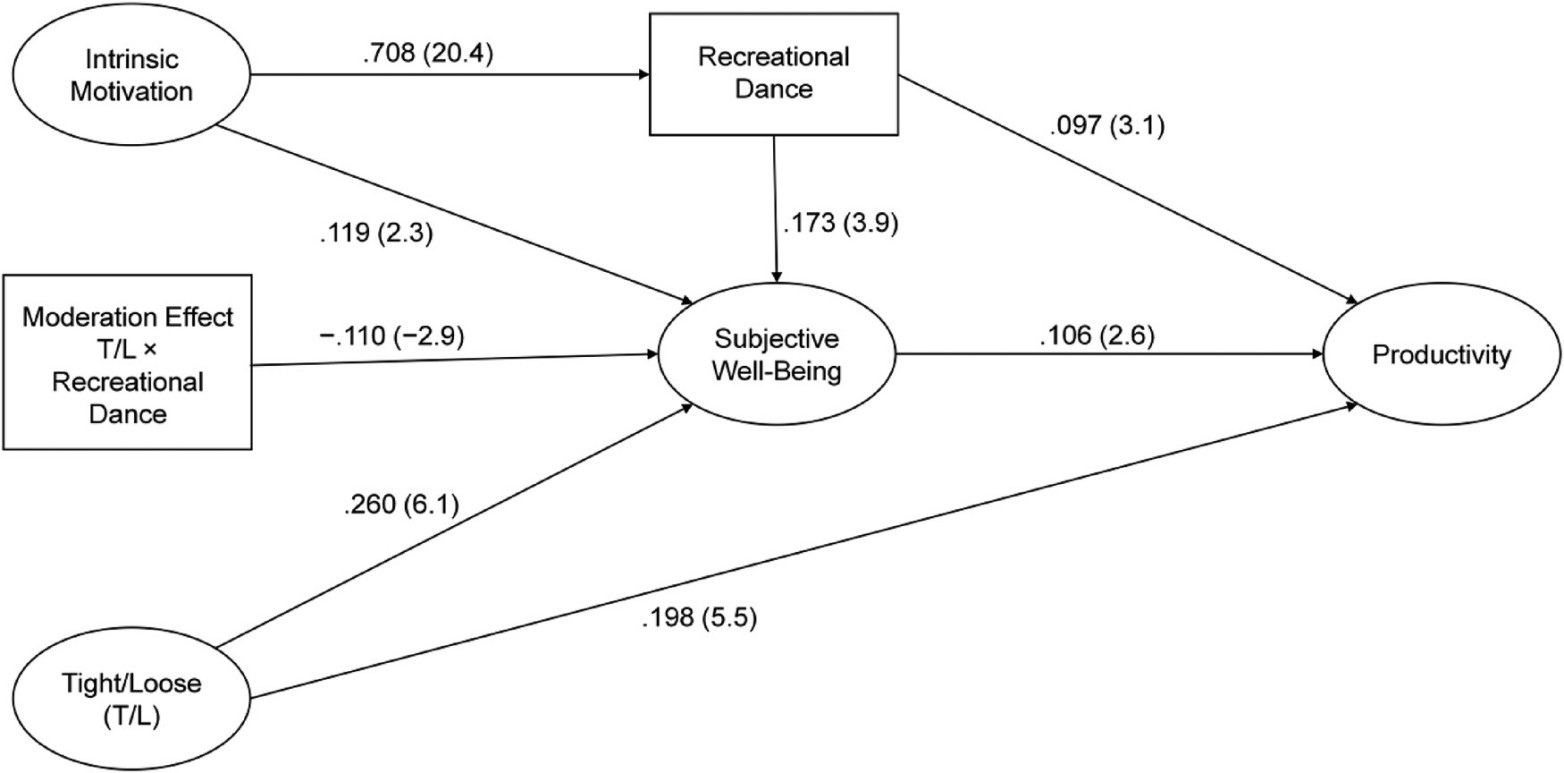
Operationalized structural equation model.

*Notes:* This figure presents standardized regression coefficients (t-values). All *p*-values < .01 except Intrinsic motivation → Well-being (<.05). Extraction: asymptotic distribution free. χ^2^ = 948, d.f. = 243; normed χ^2^ = 3.90; GFI = .930; AGFI = .910; RMSEA= .060. Tight = high, loose = low. Recreational dance: Dancer = 1. Nondancer = 0.

Two other paths that we did not hypothesize are suggested by the modification indices. Therefore, in the interest of modeling the data as thoroughly as possible, we investigated them in our model. First, tight (rather than loose) social norms have positive effects on productivity, consistent with the notions that tight norms increase efficiency and that, in a production context, the efficient combination of resources leads to higher productivity (Pieri, Vecchi, and Venturini 2018). Second, for completeness, we also explored whether perception of tighter social norms might suppress intrinsic motivation. We found the path to be only marginally significant (standardized weight: −.046, t = −1.8, *p* < .1). Other paths and fit statistics remained substantially unchanged when this path is included, so in the interest of brevity, we do not discuss details here and recommend this finding as a topic for further research. Figure 2 illustrates our findings.

## Discussion

### Theoretical Contributions

Our analysis of the impact of recreational dance on consumers’ SWB and their perceived productivity makes several theoretical contributions. First, we find that recreational dance has a direct positive impact on both constructs. Specifically, our results demonstrate that recreational dancers are more productive than nondancers because of their higher well-being levels. In addition, recreational dancers are more productive than nondancers, independent of their level of well-being, which we suggest may be due to greater cognitive skills. Our analysis also uncovers another interesting relationship between intrinsic motivation and productivity: an effect that is mediated by recreational dance and SWB. This finding, to the best of our knowledge, is unique in the relevant literature.

Second, the relationship between recreational dance and SWB is stronger when social norms are perceived as weaker. This finding is reasonable, considering that since the COVID-19 pandemic, lockdowns and social distancing rules have restricted the number of recreational activities that people can engage in. Despite the large uptake of online exercise classes and outdoor sports (Griddle 2020), our results suggest that online dance classes might not be a perfect substitute for face-to-face sessions. Thus, the well-being benefits of this activity could be better realized in looser cultures. Nevertheless, the positive effect of dance on productivity remains, even in tight cultures.

A third finding is that perception of tighter norms is associated with higher well-being and productivity, consistent with the argument that in crisis situations, individuals’ preferences shift toward tighter social norms (Seitz et al. 2020), which are associated with higher productivity (Gelfand, Harrington, and Fernandez 2017). In addition, people in tighter cultures feel more in control of their decisions, more autonomous, and more competent, all factors that positively relate to well-being. Similarly, in countries with tighter COVID-19 rules, employees have been forced to work at home, a factor that has in many cases resulted in higher efficiency and perceived productivity (Chung et al. 2020). Thus, our results are consistent with preliminary evidence of the effect of lockdown restrictions on productivity performance. This finding is particularly important considering that during the COVID-19 pandemic governments have imposed tight measures on citizens to reduce the spread of the virus. Such measures are more difficult to implement in looser cultures, as they reinforce the negative psychological effects of the restrictions. Importantly, productivity benefits of recreational dance are valid irrespective of perceptions of tight or loose social norms, which suggests that organizations marketing recreational dance internationally can apply standardized marketing communication strategies. Finally, our study contributes to research on intrinsic motivation by confirming the positive association between self-determination and SWB (Kasser et al. 2014).

### Managerial Implications

Our results have substantial implications for international marketing practitioners across three areas.

#### Implications for organizations that run offices in multiple countries

Organizations can benefit from enhancing corporate well-being by promoting a good work–life balance for employees. The firm and its employees have a reciprocal relationship, in that both can affect and be affected by each other, which plays a key role in the success or failure of the organization (Freeman and Phillips 2002). Prior literature indicates that healthy/happy employees benefit organizations in two main ways: through a reduction of health care costs and increased productivity (Pescud et al. 2015). Our findings indicate that workers who actively participate in recreational dance experience higher SWB and are more productive at work compared with workers who engage in other forms of physical activities. Therefore, a first implication of our study is that organizations can boost their employees’ SWB and productivity by including dance classes in corporate well-being programs.

Second, the introduction of dance-based well-being programs may inspire employees to advocate for their organization (Kang and Sung 2017; Kim and Yunna 2011), a factor that plays a critical role in organizations’ future performance (Thelen 2020). Third, customers prefer to connect with businesses that show goodwill toward their employees (Yohn 2019). Therefore, corporate well-being programs that include dance can make a company more compelling in its customers’ eyes. Our results demonstrate that the benefits are valid across cultures, although the outcomes appear to be greater in countries characterized by tighter social norms.

On a wider note, we have demonstrated that self-determination and perceptions of cultural tightness (as well as subjective well-being and job productivity) are one-dimensional latent variables, indicated by parsimonious scales that are reasonably stable across countries. These scales are convenient and easy to administer. International marketing managers can utilize these variables in marketing not just dance, but other physical activities. The stability of the scales across countries is important, not least because the COVID-19 pandemic has led to many dance and exercise classes being moved online and, therefore, able to be marketed globally. Notwithstanding that dancing is restricted at the time of writing due to COVID-19 social distancing measures, solo dancing, online dance classes, and competitions have become increasingly popular. Recreational dance has a major part to play in counteracting the negative well-being effects of the pandemic, and our data show stronger effects in looser cultures.

#### Implications for dance organizations

Our results have managerial implications for dance organizations that aim to recruit members from across the world. Participants in all three countries were particularly motivated to dance by happiness, fun, and interest, in addition to autonomy, competence, and relatedness to others. Therefore, dance organizations could include these motivational factors in their marketing communications and standardize their messaging across countries. Our finding of a positive associations between dancing, well-being, and productivity can be useful for dance organizations and dance schools to promote recreational dance to employers. These firms could promote dance as a win-win employer–employee benefit strategy, thus leading to a new stream of revenues for dance organizations worldwide. Dance organizations already market themselves internationally to their members and potential new members by promoting dance education, the benefits of recreational dance, professional teaching qualifications, and both national and international dance competitions. Building on the results of this study, they can also market dance classes as a pathway to increase well-being and productivity at work. In addition, our results suggest little need to differentiate marketing communications in different countries, as the positive results apply across cultures.

#### Policy implications for public health marketing

This study suggests considerable potential for public health marketing of the benefits of recreational dance. Since the onset of the COVID-19 pandemic, well-being has gained much attention internationally. Participation in recreational dance can provide a route to increasing global well-being, especially during a crisis, regardless of differences in perceived culture. Our findings for the United Kingdom, Italy, and Brazil may generalize to other countries, and international marketers can promote recreational dance as a route to increasing well-being and productivity at work across cultures and borders, which is of interest to organizations such as the WHO, which works internationally to promote health,^4^ and the Organisation for Economic Co-operation and Development, which aims to promote sustainable growth internationally.^5^

Research shows ample evidence that higher SWB is associated with better health and lower mortality (e.g., Acosta-González and Marcenaro-Gutiérrez 2021; Liu et al. 2016), a relationship that holds across cultures (Ngamaba, Panagioti, and Armitage 2017). Lack of exercise increases the risk of death by approximately 25% compared with people who exercise for 150 minutes per week, and it is one of the top ten risk factors worldwide, responsible for 3.2 million deaths per year (Mendis et al. 2014). In the three countries in our study, public health marketing is a major concern, as approximately one-third of adults do not get sufficient exercise (Jaime et al. 2013; Mendis et al. 2014; Ritchie and Roser 2017). In our analysis, motivation to perform physical activity is lowest in the United Kingdom compared with the other two countries, which could indicate that international marketers should provide extra motivation to U.K. consumers when promoting health benefits of physical exercise. Our results also reveal that recreational dancers’ well-being is highest in the United Kingdom compared with the other two countries. Therefore, marketing recreational dance could serve as a route to motivating people in the United Kingdom to exercise more, thereby improving well-being and health and possibly reducing health service costs.

## Limitations and Future Research Directions

Multiple alternative mechanisms may influence the relationships between well-being and productivity. For example, diminishing distracted worrying via a positive happiness shock leads to more effort at work (Oswald, Proto, and Sgroi 2015). Interpersonal processes in which well-being encourages individuals to help coworkers also have beneficial effects on productivity (Tsai, Chen, and Liu 2007). Finally, another important mechanism is the effect of dance on (especially mental) health and cognition (Ford et al. 2011). Drawing from the neuroscience literature, we refer to cognition to explain the direct relationship between recreational dance and productivity. However, other mechanisms could also be important. For example, scholars generally agree on the negative relationship between cultural tightness and creativity (e.g., Chua, Roth, and Lemoine 2015). Creativity may be a key variable influencing our model, as it can promote innovation and lead to higher productivity performance. Our data do not have all the necessary information to expand our model and consider these additional mechanisms, but we believe that they are important developments for future research.

Another limitation of our study relates to the lack of information on the type of physical activities among the control group, which prevents us from distinguishing between the benefits of recreational dance from the benefits of any other physical exercise. Nevertheless, our analysis demonstrates that recreational dancers experience higher levels of well-being and they are more productive in their workplace, compared with nondancers. Although this study provides relevant initial empirical generalizations on the use of recreational dance for well-being and productivity, the data are limited to three countries that have relatively similar scores on the tight–loose continuum (Gelfand et al. 2011). Thus, extending this analysis to other countries is important, to derive stronger empirical generalizations that go beyond our initial yet novel empirical findings. Furthermore, our research findings would benefit from an alternative research strategy that would support relevance and realism of context (McGrath, Martin, and Kulka 1982). In other words, future research that allows people to interact in their natural environment, like case studies or field experiments, would enhance the rigor and the relevance of the current research.

## Supplemental Material

sj-pdf-1-jig-10.1177_1069031X221079609 - Supplemental material for Shall We Dance? Recreational Dance, Well-Being and Productivity Performance During COVID-19: A Three-Country Study Click here for additional data file. Supplemental material, sj-pdf-1-jig-10.1177_1069031X221079609 for Shall We Dance? Recreational Dance, Well-Being and Productivity Performance During COVID-19: A Three-Country Study by Michela Vecchi, Patrick Elf, Akiko Ueno, Athina Dilmperi, Charles Dennis and Luke Devereux in Journal of International Marketing

## Notes

1 Although evidence of the positive effects of physical exercise on well-being is overwhelming, it is important to acknowledge the downsides of overexercising, which can result in exercise addiction and withdrawal, a phenomenon observed particularly in women. Specifically, overexercising leads to the “‘female athlete triad,’ namely, disordered eating, amenorrhoea, and osteoporosis” (Scully et al. 1998, p. 116), often exacerbated by obsessive dieting (Benson, Engelbert-Fenton, and Eisenman 1996). Participants in activities in which low body weight is expected—including, for example, dance—are most at risk of morbidity and mortality (Otis 1998). However, in recent years, emerging evidence stressing the positive effects of dance has far outweighed the negatives.

2 For a detailed overview of this literature, see Clark (2018). Happiness has recently been advocated as a central goal for U.K. government policy (Frijters et al. 2020).

3 We extracted the values of the moderation term using SmartPLS 3.3.3 (appropriate for the two-stage approach). We performed the SEM in Amos (appropriate for models that include an endogenous dichotomous variable, namely, recreational dancers).

4 See https://www.who.int/about/what-we-do (accessed February 9, 2022).

5 https://corporatefinanceinstitute.com/resources/knowledge/economics/oecd (accessed February 9, 2022).
