# Supplementary material for: Shall We Dance? Recreational Dance, Well-Being and Productivity Performance During COVID-19: A Three-Country Study
Source: J Int Mark. 2022 Jun;30(2):56–72. doi: 10.1177/1069031X221079609 (PMC9133908; doi:10.1177/1069031X221079609)
Supplement: sj-pdf-1-jig-10.1177_1069031X221079609 - Supplemental material for Shall We Dance? Recreational Dance, Well-Being and Productivity Performance During COVID-19: A Three-Country Study [file sj-pdf-1-jig-10.1177_1069031X221079609.pdf]

*WEB APPENDIX*

**Shall We Dance? Recreational Dance, Well-Being, and Productivity Performance During COVID-19: A Three-Country Study**

Michela Vecchi (m.vecchi@mdx.ac.uk), Patrick Elf (p.elf@mdx.ac.uk), Akiko Ueno (a.ueno@mdx.ac.uk), Athina Dilmperi (a.dilmperi@mdx.ac.uk), Charles Dennis (c.dennis@mdx.ac.uk), Luke Devereux (l.devereux@mdx.ac.uk)

Middlesex University Business School

William Building

The Burroughs

London NW4 4BT

United Kingdom

**CONTENTS**

***WEB APPENDIX A*** .......... 2

***WEB APPENDIX B*** .......... 9

***WEB APPENDIX C*** .......... 10

***WEB APPENDIX D*** .......... 11

These materials have been supplied by the authors to aid in the understanding of their paper. The AMA is sharing these materials at the request of the authors.

*WEB APPENDIX A*

**Table W1: The effect of physical exercise and recreational dance on health and well-being**

| STUDY | Wellbeing aspect/objective | Type of dance/exercise | Sample | Main outcome |
|---|---|---|---|---|
| Borges Da Costa and Cox (2016) | Exploration of the benefits of circle dance as a human occupation. | Circle dance performed regularly (no information about frequency). | 39 participants of the age of 18, already practicing the dance. | Dance provides a sense of fulfilment and contributes to well-being and flow. It builds a sense of community. |
| Colcombe and Kramer (2003) | Meta-analysis. Examining the hypothesis that aerobic fitness enhances the cognitive vitality of healthy but sedentary older adults. | Aerobic fitness. | 18 studies. | Fitness training benefits executive control processes. Effects on cognition are moderated by a number of factors e.g. length of fitness training, type of intervention, duration of training session and gender. |
| Conner, Patterson-Price, and Faulkner (2020) | Investigation of the psychological and physical health benefits of cultural dance among dancers. | West African dance. | Semi-structured interviews of 36 dance students, carried out in 2017 and 2018, aged 20-66. | Dance improves concentration and memory, mood and general health. It reduces stress and anxiety. West African dance promotes connection with African culture and wider community. It is particularly suitable in older age. |
| Earhart (2009) | Review on the benefits of dance for people affected by Parkinson Disease (PD) and healthy seniors. | Tango, specific dance programmes for PD, waltz/fox trot, Thai Chi, jazz dance, folkloristic dance. Mostly twice -weekly sessions of 60-90 minutes, over period ranging from 2 - 12 weeks. | 21 studies. | Dancers showed improvement in balance and walking speed, cognitive performance, endurance. They also revealed increased motivation to carry on dancing. |

| | | | | |
|---|---|---|---|---|
| Gerber and Pühse (2009) | Review paper. Exercise and fitness protecting against stress induced health complaints. | Exercise (daily physical activity, regular exercise, sufficient level of physical fitness). | 31 studies (27 of which were independent). | Exercise can be used as stress management strategy. Whilst exercise may not always help, additional stress was not generated by high exercise levels. |
| Hassmén, Koivula, and Uutela (2000) | Physical exercise frequency and psychological wellbeing. | Exercise as planned, repetitive, structured activity presumably with the goal of improving cardiopulmonary fitness. 2-3 time a week, at least 15-20 mins on each occasion and intensity that results in increased breathing and perspiration. | 3403 participants (1856 women 1547 men), age 25-64 (Finnish cardiovascular risk survey). | Those who exercised at least two to three times a week experienced significantly less depression, anger, cynical distrust, and stress compared to those exercising less frequently or not at all. |
| Houston and McGill (2013) | Subjective wellbeing, balance, stability, posture, participation, movement quality, motivation to dance, value of ballet to participants. | Twelve-week classical dance intervention (ballet) led by English National Ballet. | 24 participants, 60 - 82 years old, with Parkinson's condition. | Higher motivation, dance become an important part of people's life. Improvements in balance and stability but not posture. Improved fluency of movements. Improved well-being of participants. |

| | | | | |
|---|---|---|---|---|
| Kattenstroth et al. (2013) | Subjective wellbeing, cognition, intelligence, attention, reaction time, motor, tactile, and postural performance, cardio-respiratory performance. | Six month dance intervention, with a special dance programme developed for elderly people (Agilando). | 35 healthy participants, 67 - 74 years old. | Beneficial effects on posture and reaction times, cognitive, tactile, motor performance, and subjective well-being. Substantial improvement on working memory compared with control subjects. No effect on cardio-respiratory performance. |
| Kreutz (2008) | General health benefits, motivation, socialization. | Tango argentino (partnered dance), for a minimum period of 6 months. | 110 experienced tango dancers, 41.1% female, average age 37.96 years (SD=8.69). | Individual motivations and physical investments in dancing are of potential relevance to a healthy lifestyle, confirmed by dancers' continuous engagement. |
| Li et al. (2021) | Association of physical fitness with mental health in children. | Chinese National Student Physical Fitness Standard. BMI, vital lung capacity, 50m sprint, sit and reach, timed rope skipping, times sit ups 50m x 8 shuttle. | 269 (126 boys 143 girls) children form primary schools in China. | The positive association between physical fitness and mental health in children can be influenced by levels of resilience and anxiety. |

| | | | | |
|---|---|---|---|---|
| Mandolesi et al. (2018) | Review paper. Effects of physical exercise on cognitive functioning and wellbeing. | WHO definition of physical activity that is planned, structured, repetitive, and has as a final or an intermediate objective the improvement or maintenance of one or more components of physical fitness. | 149 articles. | Physical exercise determines positive biological and psychological effects that affect the brain and its cognitive functioning and promote wellbeing. |
| Muro and Artero (2016) | Mindfulness and life satisfaction (LS). | Latin dances (salsa, Latin or ballroom), performed at three dance schools, at least once a week, for recreational or social motives. | non-clinical sample of 81 women dancers, 121 non-dancers, aged 18-37. | Higher levels of mindfulness and LS in the dance practitioners. Dance explains 28% of the variation in LS. |
| Pinniger et al. (2012) | Psychological stress, anxiety, depression, life satisfaction, mindfulness. | Tango argentino (partnered dance), for a period of 6 weeks. | 21 tango dancers, 16 mindfulness practice, 31 control group, aged 18 - 80 years. 90.9% female, with symptoms of anxiety, stress, depression. | Both tango and mindfulness groups show greater decrease in levels of depression and stress compared to the control group. Tango dancers reported significantly lower levels of depression compared to mindfulness group. Improved anxiety and life satisfaction larger for tango group (although effect not statistically significant). |

| | | | | |
|---|---|---|---|---|
| Ratey and Loher (2001) | Review of the potential mechanisms by which physical activity improve brain function. | Aerobic fitness. | 75 studies. | Physical activity benefits brain function and executive cognitive processes (increase neuroelectric activity, brain volume, attention, learning and memory) and has a lasting impact on cognition. Physical exercise reduces the odds of developing dementia. |
| Rehfeld et al. (2017) | Mental well-being (hippocampal plasticity and balance). | 18 months of a specifically designed dance programme (constantly learning new choreographies) and a fitness programme (repetitive tasks such as cycling/walking). | 52 healthy participants, aged 63-80 at the beginning of the study. Full data available for 26 individuals. | Dancers showed volume increases in more subfields of the left HC and only dance led to an increase in one subfield of the fight HC, compared to the fitness group. Improvements in balance among dancers was superior compared to the fitness group. |
| Scully et al. (1998) | Review paper. Relation between physical exercise and depression, anxiety, stress responsivity, mood state, self-esteem, premenstrual syndrome, and body image. | Various e.g. non-aerobic, aerobic and anaerobic as well as durations. | 127 studies. | Exercise may be able to play a therapeutic role for a number of disorders (e.g. depression, anxiety, mood state, self-esteem and body image). Different conditions may require different exercise regimes. |

| | | | | |
|---|---|---|---|---|
| Shanahan et al. (2015) | Systematic review of the evidence of the benefits of dance for Parkinson; provide information on the features of dance interventions. | Different dance styles (tango, Irish dance, Waltz/foxtrot, modern dance) and Tai Chi. Number of classes reported ranged between 2 and 3 classes weekly. Duration of the intervention ranges between 2 weeks and 32 weeks. | 13 studies, 8 RCTS, 1-quasi-RCT, 4 cohort studies. Sample size in the studies range from 11 to 75, aged 61.6 - 74.4. | Dance improves balance, motor impairment, endurance. |
| Silverman and Deuster (2014) | Review paper. The biological mechanisms underlying the beneficial effects of physical fitness on mental and physical health. | Physical fitness. | 216 studies. | Physical fitness appears to buffer against stress-related disease, which can help with mood and well-being. |
| Stubbs et al. (2017) | Meta/analysis/systematic review. Investigate the benefits of exercise compared to usual treatment or control conditions in people with an anxiety and/or stress-related disorders. | Physical exercise (aerobic exercise). | 6 studies met the inclusion criteria from a pool of 62 full texts. Across the 6 studies 262 adults with anxiety/stress disorder were included. | Exercise is an important treatment option for those with anxiety/stress disorders. |

| | | | | |
|---|---|---|---|---|
| Teixeira-Machado and Coutinho (2018) | Systematic review of the evidence on how dance can alter brain volumes and structure, brain function psychomotor adjustment and levels of neurotrophic factors. | Social dance, ballroom dance (salsa, rumba, waltz, cha-cha, blues, jitterbug, tango), special dance programme (Agilando). Practice taking place mostly twice weekly. Various controls based on walking, aerobic exercises, playing instruments, health education. | 8 studies, all RCTs, including 889 participants aged between 60 and 94 years of age. With the exception of 1 study, all the others focused on healthy seniors. | Increase in hippocampal volume, additional to health fitness. Impact on white matter with effects on learning and cognitive level. Improvements in attention, cognitive speed, executive functions and memory, balance. |
| Wyss et al. (2016) | The role of physical fitness levels on the autonomic nervous system responses to acute psychosocial stress. | Swiss Army physical fitness test battery. Progressive endurance run, standing long jump, seated shot put, trunk muscle strength test, one leg standing test. | 302 male recruits during first week of Swiss Army basic training. | Good physical fitness, especially good aerobic endurance capacity, is an important protective factor against health-threatening reactions to acute psychosocial stress. |
| Zubala et al. (2017) | Review paper. Promotion of physical activity interventions for community dwelling older adults. | Different types of physical activity including walking, gardening, dance, low to moderate exercise, tai chi, aquatic exercises, yoga, jogging. | 19 studies. | Interventions to promote physical activity among older adults are generally effective but there is uncertainty around the most beneficial intervention components. |

*WEB APPENDIX B*

**Table W2: Scale items included in the study.**

| Well-being (answers 1-7, 1 = strongly disagree, 7 = strongly agree) | Loading |
|---|---|
| In most ways my life is close to my ideal. | .798 |
| The conditions of my life are excellent. | .812 |
| I am satisfied with my life. | .889 |
| I have the important things I want in life. | .737 |
| If I could live my life over, I would change almost nothing. | .713 |
| **Productivity (presenteeism) (answers 1 – 5, 1 = all the times, 5 = none of the times). In the past week:** | |
| How often did you do no work at times when you were supposed to be working? | .505 |
| How often did you find yourself not working as carefully as you should? | .653 |
| How often was the quality of your work lower than it should have been? | Dropped |
| How often did you not concentrate enough on your work? | .831 |
| How often did health problems limit the kind or amount of work you could do? | .686 |
| **Intrinsic motivation (answers 1 – 7, 1 = strongly disagree, 7 = strongly agree). I exercise/dance:** | |
| Because I want to be physically fit. | .615 |
| Because it is fun. | .891 |
| Because I like engaging in activities which physically challenge me. | .833 |
| Because I want to obtain new skills. | .871 |
| Because I want to improve existing skills. | .871 |
| Because I like the challenge. | .758 |
| Because it makes me happy. | .911 |
| Because I want to have more energy. | Not used |
| Because I want to improve my appearance. | Not used |
| Because I think it’s interesting. | .878 |
| Because I want to meet new people. | Not used |
| Because I want to maintain my physical health and well-being. | Not used |
| Because I want to improve my body shape. | Not used |
| Because my friends want me to. | Not used |
| Because I enjoy spending time with others doing this activity. | Not used |
| **Perception of tight/loose social norms (answers 1 – 7, 1 = strongly disagree, 7 = strongly agree)** | |
| In my country, there are very clear expectations for how people should act in most situations. | .639 |
| There are many social norms that people are supposed to abide by in my country. | .737 |
| People agree upon what behaviors are appropriate versus inappropriate in most situations in my country. | .816 |
| People in my country have a great deal of freedom in deciding how they want to behave in most situations (7) | Dropped |
| In my country, if someone acts in an inappropriate way, others will strongly disapprove. | Dropped |
| People in my country almost always comply with social norms. | .644 |

## *WEB APPENDIX C*

## Table W3: Demographic characteristics of the sample (% except for the first row)

| | Dancers | Non-Dancers |
|---|---|---|
| Number of observations | 537 | 956 |
| UK | 46.39 | 53.61 |
| Italy | 34.61 | 65.39 |
| Brazil | 26.29 | 73.71 |
| Female | 87.45 | 85.81 |
| Male | 11.24 | 13.76 |
| Other | 1.31 | 0.44 |
| 18-24 | 30.23 | 35.66 |
| 25-44 | 28.33 | 35.50 |
| 45-59 | 27.00 | 23.90 |
| 60 + | 14.45 | 7.85 |
| No formal education | 0.56 | 2.07 |
| Primary | 2.79 | 2.94 |
| Secondary | 40.22 | 48.2 |
| Tertiary | 56.42 | 46.78 |

## *WEB APPENDIX D*

## Table W4: Correlations Between Constructs.

| | Construct Reliability | Average Variance Extracted | Well-being | Intrinsic Motivation | Tight/Loose | Productivity |
|---|---|---|---|---|---|---|
| Well-being | 0.903 | 0.651 | **0.807** | | | |
| Intrinsic motivation | 0.953 | 0.718 | 0.297 | **0.847** | | |
| Tight/Loose | 0.817 | 0.529 | 0.351 | 0.190 | **0.727** | |
| Productivity | 0.845 | 0.529 | 0.165 | 0.058 | 0.207 | **0.727** |

Note: Figures in the diagonal are the square roots of the average variance extracted
